# Clinical application of concentrate growth factors combined with bone substitute in Alveolar ridge preservation of anterior teeth

**DOI:** 10.1186/s12903-022-02091-8

**Published:** 2022-03-03

**Authors:** Dilinuer Keranmu, Nijiati Nuermuhanmode, Ailimaierdan Ainiwaer, Guli, Dilidaer Taxifulati, Wang Shan, Wang Ling

**Affiliations:** Outpatient Department of Oral Surgery, The First Affiliated Hospital of Xinjiang Medical University (Affiliated Stomatological Hospital). Research Institute of Stomatology of Xinjiang Uygur Autonomous Region, No.393, Xinyi Road, Xinshi District, Ürümqi, 830054 Xinjiang China

**Keywords:** Alveolar bone, Alveolar ridge preservation (ARP), Concentrate growth factors (CGF), Bio-oss bone powder

## Abstract

**Objective:**

To investigate the clinical effect of concentrated growth factors (CGF) combined with deproteinized bovine bone mineral (DBBM) on Alveolar ridge preservation during implantology.

**Methods:**

A total of 38 patients were selected and randomly divided into 2 groups, with 19 cases in each group. The extraction sockets were filled with DBBM with or without CGF. Visual analogue scale (VAS) pain score was recorded within1 week and Landry wound healing index (LWHI) was recorded at 1, 2 and 3 weeks after operation. CBCT was taken preoperatively and 3 and 6 months postoperatively to measure and compare the changes of vertical height, width and gray value of alveolar bone at extraction site. The changes of alveolar bone contour were observed clinically and compared between the two groups.

**Results:**

The VAS score of CGF group was lower than control group on the 1st and 3rd day after operation (*P* < 0.05). The LWHI of CGF group was higher than control group 1 week after operation (*P* < 0.05). The absorption of the labial and palatal plates height and the width in the CGF group was significantly less than the control group at 3 months (*P* < 0.05). The gray value of alveolar bone in CGF group was significantly higher than control group at 3 months (*P* < 0.05). There was no significant difference in new bone contour between the two groups (*P* > 0.05). 94.7% cases in CGF group did not undergo bone grafting, which was significantly higher than control group (78.9%).

**Conclusions:**

The use of CGF combined with DBBM can help to reduce postoperative pain at the early stage of healing, form sufficient keratinized gingival tissue, effectively maintain the height and width of alveolar bone in the three-dimensional direction and provide good conditions for implant repair in the future.

## Background

Implant restoration has been gradually accepted by more and more patients, because there is no damage to the adjacent teeth. Besides, implants have the same function as natural teeth which can bear and transmit masticatory force well. However, the absorption of alveolar bone after tooth extraction limits the application of implant. Alveolar bone can be absorbed to different degrees after tooth extraction, especially after 3–6 months. The alveolar bone can be absorbed to varying degrees 3–6 months after tooth extraction, and most of dimensional change can be occurred within 2 weeks [1]. The alveolar ridge could lose 29–63% (2.46–4.56 mm) of its original width and 11–22% (0.8–1.5 mm) of its original height [2], which will lead to the deficiency of alveolar ridge bone and affect the long-term use and aesthetic effect of implants. Therefore, how to preserve the mass of alveolar bone is the critical problem of implant repair [3].

The concept of Alveolar ridge preservation (ARP) was first proposed in 1994. It refers to the protective intervention on the sites that need delayed implant restoration while extracting the tooth [4]. The morphology of soft and hard tissue can be preserved to the greatest extent by reducing the bone loss of extraction socket and accelerating bone regeneration. There are many different ARP techniques and various types of materials, such as autogenous bone [5], allografts [6], xenografts and platelet concentrates [7, 8]. The main goal of the bone graft material is to serve as a scaffold and maintain a space for bone ingrowth, blood vessels formation, to support soft tissues and to improve the quality and quantity of regenerated bone [9]. DBBM is a classical xenogeneic bone graft material, which is made from deorganized bovine limb bone, and generally biocompatible and structurally similar to human bone. DBBM has been developed as the preferred alternative bone material and used in restoration of bone defects around implants, ridge preservation after extraction [10], maxillary sinus augmentation [11], treatment of cyst and generally resulted in new attachment and cementum formation when compared to ungrafted sites.

In recent years, concentrated platelets have been used in wound healing because of their high growth factor content. Among the preparations, CGF, platelet-rich plasma (PRP), platelet-derived growth factor, transforming growth factor beta and platelet-rich fibrin (PRF) currently used for the regeneration and reconstruction of bone and connective tissues are. CGF is a new generation of plasma extract prepared from patients' own venous blood by special centrifugation. CGF was first proposed by Sacco in 2006, which contains high concentrations of a variety of growth factors and fibrin [12]. The preparation process is simple, without the risk of cross infection and allergic reaction, it is safe and reliable for clinical use. CGF has been widely used in the fields of oral implantation, maxillary sinus lifting, treatment of jaw cysts and promotion of fracture healing [13–16].

However, the researchs on the combined application of CGF with DBBM in ARP were limited. Therefore, the aim of this study was to perform a clinical and radiographic evaluation of the ARP technique using CGF and DBBM, to observe the conditions of the alveolar ridge bone after tooth extraction and evaluate the application value of CGF combined with Bio-oss bone powder in alveolar bone increment.

## Materials and methods

### Patient population and enrollment

38 patients who underwent incisor, lateral incisor and canine single tooth extraction at the Outpatient department of Stomatology from October 2020 to May 2021 were collected. 38 extraction sites were randomly divided into CGF group and control group, 19 cases in each. All patients were informed about the potential benefits and risks of surgery, as well as alternative treatment options, and volunteered to participate in and signed an informed consent. The protocol of this study was consistent with the ethical guidelines of the Declaration of Helsinki and was approved by the Ethics Committee of the First Affiliated Hospital of Xinjiang Medical University (Ethics approval No.: 210723-08). All patients voluntarily participated in the study and signed informed consent.

Inclusion criteria were as follows (1) Patients over 18 and under 35; (2) Teeth cannot be retained due to severe caries, chronic periapical periodontitis or trauma, and need implant restoration; (3) All extraction sites had adjacent teeth with healthy periodontal tissue or only mild periodontal disease (plaque index and bleeding score less than 15%); (4) At least 2 or more bone plates exists at the extraction site; (5) Without serious systemic diseases, psychosis and epilepsy; (6) Non smoking and good compliance.

Exclusion criteria were as follows (1) Patients with acute periodontal or periapical infection; (2) Both of the buccal and lingual alveolar bone resorption exceeds 25% of root length; (3) With severe hypertension, diabetes, kidney and liver diseases, especially patients taking anticoagulants; (4) Pregnant patients; (5) Patients with a history of radiotherapy and chemotherapy.

### Preoperative work-up

**Fig. 1:**
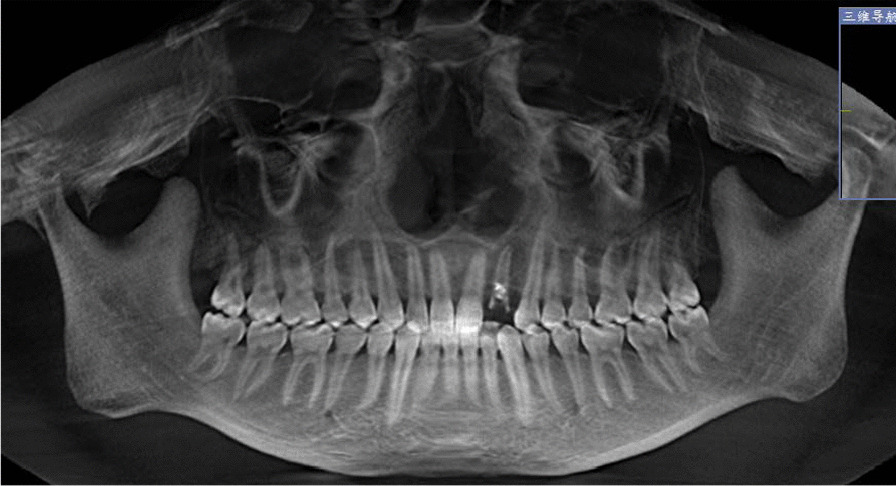
CBCT image of CGF group before tooth extraction

**Fig. 2:**
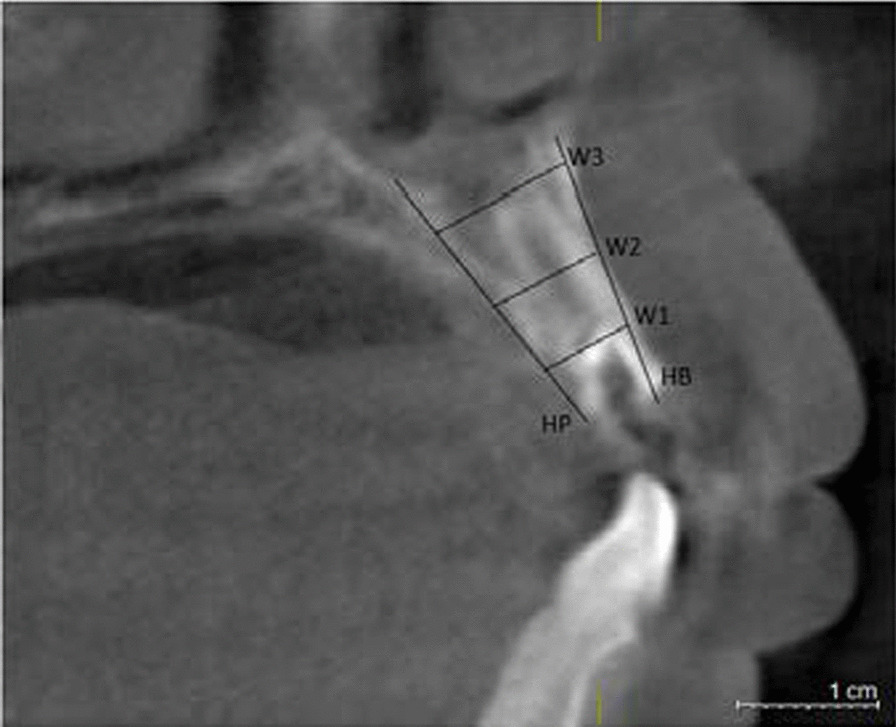
The radiographic landmarks used for measurement of bone width and bone height on CBCT images

Preoperative examination including general conditions, routine blood tests, oral hygiene, occlusal relationship, etc. CBCT was taken to measure the height and width of alveolar bone. (Fig. 1). We adjusted the sagittal and coronal three-dimensional positions, selected the plane passing through the center of the tooth extraction site, marked the line passing through the long axis of the affected tooth, selected the apex of the palate or the lower edge of the mandible as the fixed reference point, and identified the buccal bone plate and palatal plate accordance with the long axis of the affected tooth as Hb and Hp respectively. Vertical resorption included both the buccal side (Hb) and the palatal/lingual side (Hp). The width of alveolar ridge was measured at 3,8, and 12 mm relative to the alveolar bone crest, which were recorded as W1, W2 and W3 respectively (Fig. 2) [17]. Grayscale was measured in the site preservation area, that is, in the center of tooth socket extraction, the same area was intercepted and the gray level is measured, the average value is taken after three repetitions. The table of Breakdown with tooth type and sample distribution are presented in Table 1.

**Table 1:** Breakdown with tooth type and sample distribution

FDI dental representation						
Case (Maxillary)	3	4	2	2	4	3
ISO-3950	13	12	11	21	22	23
	43	42	41	31	32	33
Case (Mandible)	2	5	2	2	5	4

Case: Represents the number of cases per tooth position

FDI: International common tooth counting method, ten represents the quadrant, each represents the tooth position

**Fig. 3:**
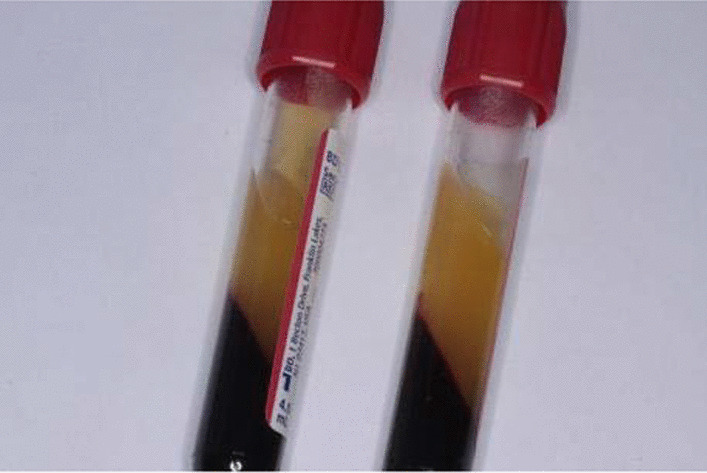
The CGF after separation

*Preparation of CGF* Venous blood was collected from each patients in CGF group using sterile vacuum tubes (Greiner Bio-One, GmbH, Kremsmunster, Austria), without additive. Then the tubes with whole blood (4 mL in each) were immediately centrifuged by Medifuge (Silfradent, Italy) at fixed temperature. After centrifugation, CGF gel represented as the buffy coat in the middle layer and was carefully isolated from the red blood cell clots (Fig. 3). One of the prepared CGF was pressurized to remove the liquid components to make CGF membrane for later use.

### Surgical procedure

**Fig. 4:**
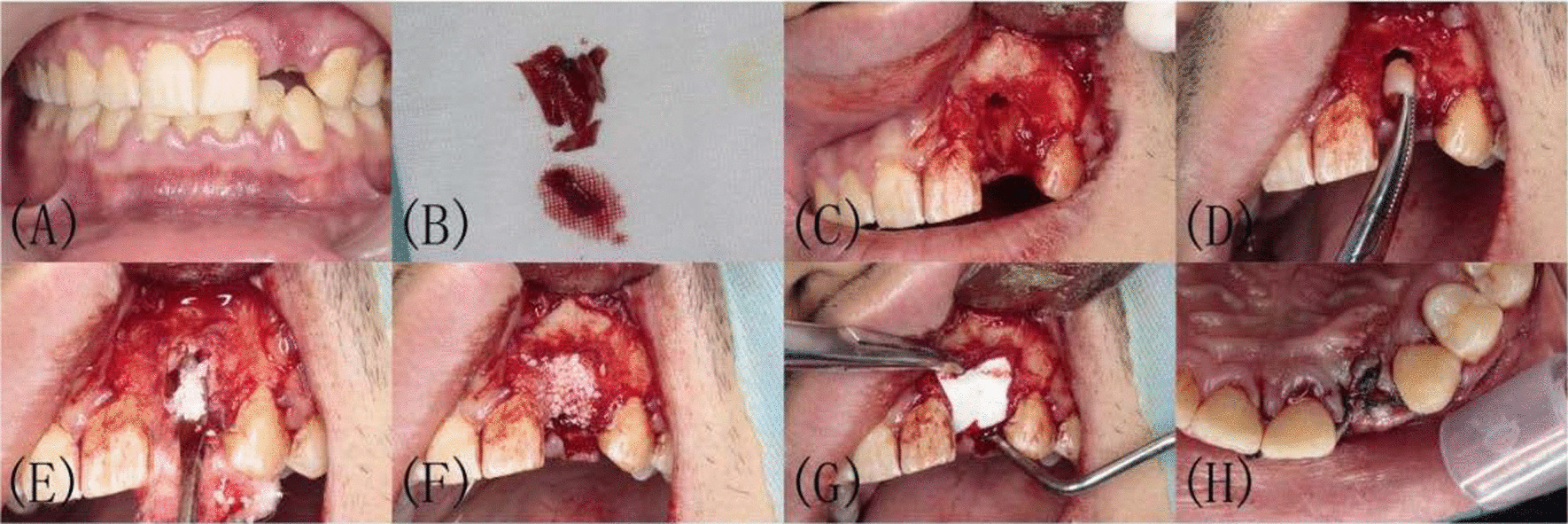
Surgical procedure of CGF group. **A** Labial view before tooth extraction. **B** Extraction and ebridement. **C** Extraction sockets after scoraping. **D**, **E** Small pieces of CGF mixed with Bio-oss. **F** Filled with mixture of CGF and Bio-oss. **G** Socket covered with CGF membrane and then collagen membrane. **H** Contralateral suture of gingival flap

Mouth rinsing was performed 3 times with 0.2% chlorhexidine solution before operation. Under local infiltration anesthesia with articaine, the gingival were separated and the affected tooth was extracted atraumatically. After removing the affected tooth, the periapical lesion was curetted by using bone curettes when the root tip was inflamed and the surgical area was then rinsed with physiological saline. In the CGF group, CGF was cut into small particles, fully mixed with DBBM (Bio-Oss; Geistlich, Pharma AG, Wolhusen, Switzerland), and filled into the extraction socket in layers to make it about 2 mm higher than the crest of the surrounding alveolar ridge, the wound was completely covered with CGF membrane and then Collagen membrane (Bio-Gide; Geistlich, Pharma AG, Wolhusen, Switzerland). Finally, the flap was repositioned coronally and sutured tightly with non-resorbable sutures. In the control group, Bio-Oss was filled into the extraction socket to make it about 2 mm higher than the crest of the surrounding alveolar ridge, the wound was completely covered with Collagen membrane and the flap was sutured (Fig. 4).

### Postoperative examination and treatment

After the grafting surgery, systemic antibiotics were prescribed to all patients 1 day before operation for 5 days. Visual analogue scale (VAS) was used to measure the degree of postoperative pain on day 1, 3, and 7. The sutures were removed after 1 week. According to the color of the gingiva, the presence or absence of granulation tissue, bleeding, pyorrhea and epithelial formation, the Landry wound healing index (LWHI) was recorded at 1, 2 and 3 weeks. [18] CBCT was taken after 3 and 6 months to compare the changes of alveolar bone height, width and gray value of socket between the two groups. The contour of alveolar bone was recorded and compared with the adjacent teeth and the same teeth on the other side after 3 and 6 months.

### Statistical analysis

Measurements were recorded in a spreadsheet in Excel 2013 (Microsoft Corporation, WA) and were then analyzed using SPSS 20.0 (SPSS Inc., IL). Data are given as mean ± SD and were evaluated via a Shapiro–Wilk test to assess distribution normality. Normally distributed data were compared via t-test or analysis of variance (ANOVA), while non-normally distributed data were compared via Mann–Whitney U test. A significance level of α = 0.05 was used for all analyses.

## Results

### General observation

A total of 38 patients who met the inclusion criteria were identified in this study, 19 cases in each group, 23 females and 15 males, with a mean age of (28.89 ± 2.7) years, all of them completed the experimental protocols and subsequent implant restoration. All sockets healed uneventfully, and no adverse reactions such as wound dehiscence and acute infection were observed during the 6-month clinical healing period. The contour of alveolar bone in the CGF group was more conducive to implant implantation. There was no significant differences in average patient age between the two groups.

### Postoperative pain and soft tissue healing

The VAS score of CGF group was lower than control group on the 1st day after operation (*P* < 0.05) (Table 2). The LWHI of the CGF group and the control group at 1, 2 and 3 weeks after operation is shown in Table 3. The LWHI of CGF group was higher than control group 1 week after operation (*P* < 0.05).

**Table 2:** VAS scores of the two groups

Group	Visual analogue scale (VAS)		
	1 day	3 days	7 days
Control Group	5.61 ± 0.85	3.03 ± 0.61	1.17 ± 0.99
CGF Group	4.33 ± 1.07	3.00 ± 0.62	1.17 ± 0.99
*P* value	< 0.05	0.893	1

**Table 3:** LWHI of the two groups

Landry wound healing index (LWHI)			
Group	1 week	2 weeks	3 weeks
Control group	2.50 ± 0.62	3.88 ± 0.58	4.83 ± 0.38
CGF group	3.94 ± 0.64	4.83 ± 0.38	5.00 ± 0.00
*P* value	< 0.05	0.092	0.074

### CBCT analysis

**Fig. 5:**
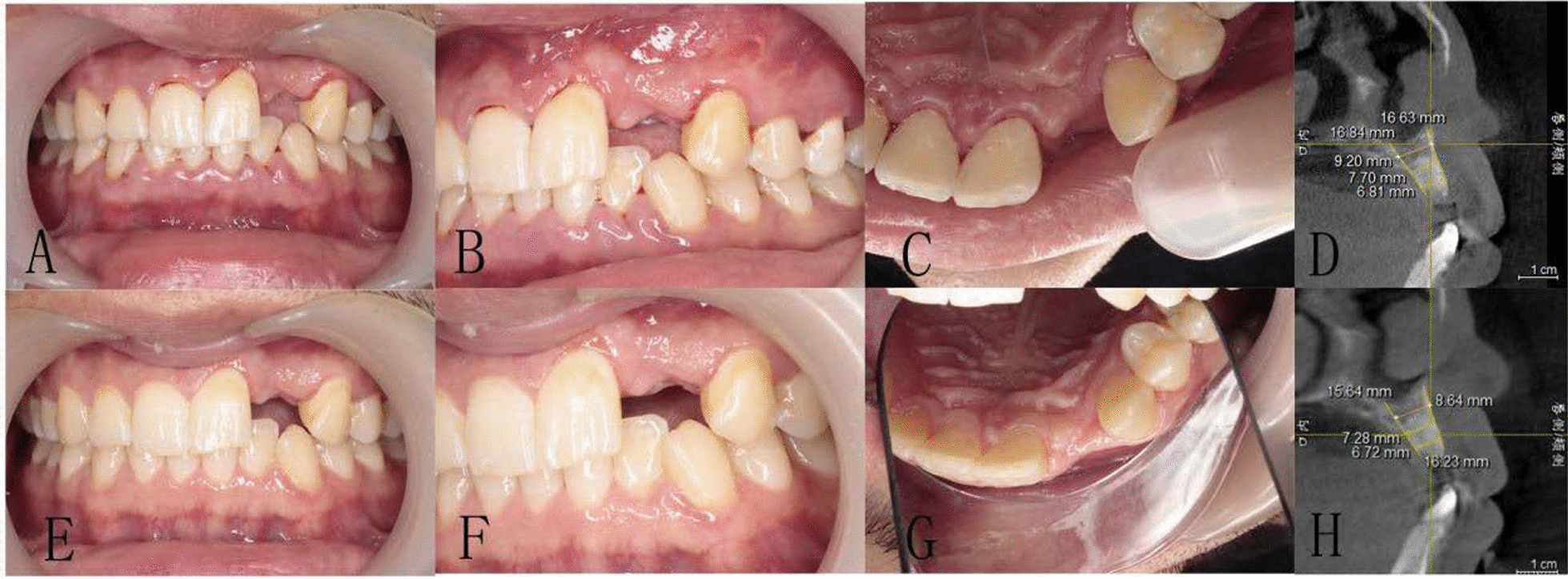
Clinical view and CBCT images of CGF group at 3 and 6 months after operation. **A**–**D** Intraoral and CBCT image of CGF group 3 months after operation. **E**–**H** Intraoral and CBCT image of CGF group 6 months after operation

**Fig. 6:**
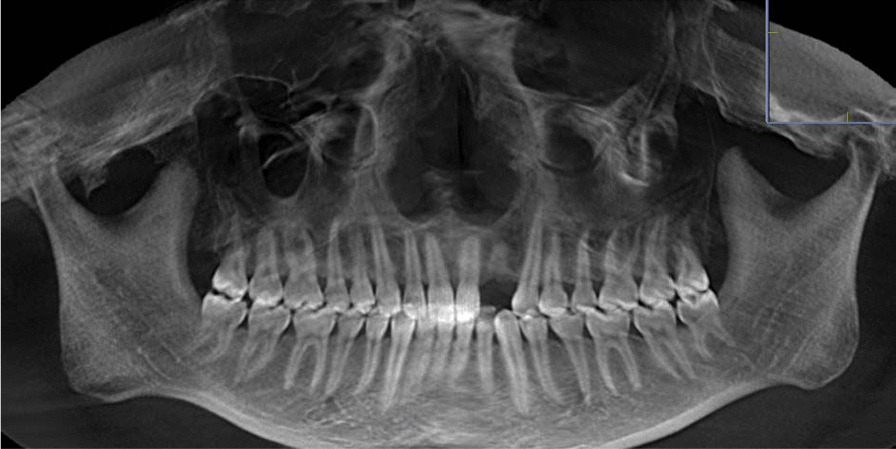
CBCT images of CGF group 6 months after operation

The buccal and palatal/lingual absorption of the two groups at 3 and 6 months after tooth extraction is shown in Table 4. The absorption of the buccal and palatal/lingual plates height and the and changes in the two groups ridge width in the two groups at 3 and 6 months after tooth extraction is shown in Table 4. There were significant differences in buccal and palatal/lingual vertical bone resorption and the ridge width between the two groups at 3 and 6 months after operation (*P* < 0.05). The gray value of alveolar bone in CGF group was significantly higher than control group at 3 months (*P* < 0.05). (Table 5, Figs. 5, 6).

**Table 4:** The changes of ridge height, width and grey value between preoperative, 3 and 6 months later in two groups

Items	Group		*P* value
	Control group	CGF group	
Hb(mm)			
Preoperative	17.22 ± 1.13	17.16 ± 1.25	0.868
3 M	15.66 ± 1.34	16.86 ± 1.25	< 0.05
6 M	14.89 ± 1.53	16.52 ± 1.26	< 0.05
Hp(mm)			
Preoperative	16.85 ± 1.34	16.68 ± 1.36	0.713
3 M	14.01 ± 1.36	16.61 ± 1.37	< 0.05
6 M	13.20 ± 1.25	15.18 ± 1.37	< 0.05
W1(mm)			
Preoperative	6.37 ± 0.76	6.41 ± 0.60	0.866
3 M	4.43 ± 0.67	5.91 ± 0.67	< 0.05
6 M	3.22 ± 0.69	5.10 ± 0.71	< 0.05
W2(mm)			
Preoperative	8.10 ± 0.60	8.26 ± 0.55	0.432
3 M	5.49 ± 1.05	7.60 ± 0.74	< 0.05
6 M	4.82 ± 0.99	6.93 ± 0.71	< 0.05
W3(mm)			
Preoperative	10.76 ± 0.60	10.94 ± 0.51	0.32
3 M	9.10 ± 0.86	10.20 ± 0.59	< 0.05
6 M	8.51 ± 0.91	9.46 ± 0.43	0.28

3 M represents 3 months after operation and 6 M represents 6 months after operation

**Table 5:** The gray value of alveolar ridge were compared by CBCT at 3 and 6 months

Items		Hu	
Group	Preoperative	3 Mouths	6 Mouths
Control group	1363.72 ± 38.21	2055.67 ± 120.82	2182.00 ± 109.78
CGF group	1360.22 ± 53.04	2270.89 ± 42.29	2289.11 ± 39.01
*P* value	0.822	< 0.05	0.29

### New bone contour evaluation

Clinical observation showed that the attached gingival of the extraction site in the CGF group was sufficient, with normal color and plump appearance, and no infection or necrosis was observed. All the implants were successfully completed. In the control group, the extraction site healed well, but the alveolar crest showed different degrees of atrophy, and the attached gingival was relatively narrow. There was no significant difference in new bone contour between the two groups.

### Bone grafting at the extraction site

After 6 months of follow-up, while only 1 case in the CGF group underwent bone increment by GBR, 3 cases in the control group underwent bone increment and 1 case received connective tissue transplantation. 94.7% cases in CGF group did not undergo bone grafting, which was significantly higher than control group (78.9%).

## Discussion

The alveolar ridge resorption and soft tissue shrinkage after tooth extraction are the main problems affecting the aesthetics and function of alveolar ridge. In order to achieve successful implantation and long-term effect, sufficient bone volume, keratinized gingiva and appropriate occlusal relationship should be obtained. Therefore, alveolar bone should be preserved as much as possible during tooth extraction to reduce edentulous ridge resorption rate and promote the bone remodeling of alveolar bone [19]. Compared with the natural healing, ARP has significant advantages in preserving the size and contour of alveolar bone, especially in the maxilla [20].

Most studies on the dimensional changes of soft and hard tissues after tooth extraction suggested that the ideal bone graft material should not only have osteoconductive properties but also promote osteoinduction and osteogenesis [21]. Only autologous bone has these three characteristics and is still considered to be the gold standard for bone augmentation surgery [22]. However, due to the additional site and prolonged time of operation, the morbidity of donor side, limited autogenous bone availability and postoperative discomfort, bone substitutes are more commonly used for bone regeneration. Bone graft materials are chosen based on their ability to serve as a scaffold, maintain space for new bone ingrowth and possess osteoconductive activity [23].

DBBM has strong hydrophilicity, high biocompatibility and good plasticity, it can promote the adhesion of osteoblasts to bone, plays important role as a scaffold, and has a very low risk of causing host allergy, inflammation and immune reactions. Twenty-seven patients were randomized into two treatment groups following single tooth extraction in the incisor, canine and premolar area. In the test group, the alveolar socket was grafted with Straumann Bone Ceramic (SBC), while in the control group, Bio-Oss was applied. 8 months later, they found that both materials preserved the mesio-distal bone height of the ridge [24]. In another study, Kim et al. [25] treated the extraction socket sites of 20 first molars in two different ways, filling Bio-oss with gelatin sponge and natural healing respectively. After 3 months, the absorptivity of alveolar ridge width was 14. 3% and 20. 7% (*P* < 0. 05). Histological studies have found that the rate of new bone formation is about 26.0% ± 23.7% when DBBM was used alone, and it can even reach 48.3% [26, 27]. Although DBBM has been proven to have reliable osteogenesis effect as a bone substitute material [28], DBBM lacks osteogenesis and osteoinduction. Research confirmed that the part where DBBM directly contacts the bone surface will first lead to the formation of new bone, while it takes one year at the peripheral part without direct contact [29]. The use of DBBM alone will delay bone healing and prolong osteogenesis time to a certain extent [30]. Therefore, it is significant to explore a material to promote the osteoinduction of bone substitute materials.

CGF is a new generation of platelet concentrate after PRP and PRF. The production of CGF requires variable speeds to separate blood cells from fibrin-rich blocks, which are denser and contain a higher concentration of growth factors than PRF [16]. This results in a better regenerative capacity and greater versatility. CGF contains a large amount of platelet-derived growth factor (PDGF), vascular endothelial growth factor (VEGF), epidermal growth factor (EGF), insulin-like growth factor (IGF), fibroblast growth factor (FGF), bone morphogenetic proteins (BMPs) and metastatic growth factors-β (TGF-β) etc. Among which, FGF can accumulate macrophages, fibroblasts and other cells to the wound site through chemotaxis, thereby promoting wound healing [31, 32]. BMP can mediate osteogenesis alone and promote the formation of bone matrix and form calcified bone tissue when mix with other bone growth factors. TGF-β, as an important regulatory factor in the process of bone formation and remodeling, controls inflammation through synthetic fibrous connective tissue and local vascular proliferation, and also induces regeneration of alveolar bone [33].

In this study, none of the patients experienced rejection or wound infections around the grafting region, which indicated that the deproteinized bovine bone and CGF were safe and biocompatible. Due to the dispersive nature and small particles of DBBM, leakage often occurs, so it is necessary to cover the wound with a collagen membrane, and the gingival flap is tightly sutured to prevent the leakage of bone powder. In this study, CGF was cut into small pieces and fully mixed with DBBM, which was easy to mold. In the meantime, because of the elasticity and adhesion of CGF membrane, leakage rarely occurs even if the tooth extraction wound cannot be closed tightly.

The vertical bone loss of labial buccal bone wall was more obvious than the lingual bone wall due to its thin thickness after tooth extraction [34]. Similar conditions were observed in our study, so the alveolar crest height was divided into labial and palatal plate height, which were measured and compared respectively. Different from the changes in the control group, the height of labial and palatal plate in CGF group did not change significantly 3 months after operation. Although the width of alveolar ridge decreased, the change was much less than control group. During the implantation operation, some bone powder particles at the tooth extraction in CGF group were seen surrounded by new bone. Furthermore, CBCT showed obvious bone trabecular formation in the operation area. It can be concluded that the use of CGF combined with DBBM for ARP can effectively maintain the volume of the alveolar bone, significantly promote the regeneration of the alveolar tissue, and reduce bone resorption effectively. Therefore, when ready to insert dental implant, the CGF group has better alveolar ridge condition, more bone volume, and better surgical environment. Many other studies have shown that CGF can accelerate bone healing [14, 16, 35–37]. Among them, Kim et al. [14] applied CGF in sinus augmentation without any graft materials and confirmed respectively that CGF was effective in promoting healing of bone and can induce new bone formation rapidly. Our previous research showed that the application of CGF in recipient site with a small area of chronic periapical lesions can accelerate the regeneration of alveolar bone and the healing of inflammation, greatly shorten the healing period [35]. Fang et al. [36] found that the combinational use of CGFs with DBBM could promote new bone regeneration without adding exogenous stem cells in bilateral maxillary sinus floor augmentation, which yields effects similar to combining BMSCs with DBBM. Furthermore, Durmuş lar et al. [37] found that the combined use of CGF and bone graft enhanced the expression of osteogenic related genes and stem cell marker STRO-1, and promoted bone regeneration of large defects around implants (about 2.37 mm in diameter). Together, these results recommend the use of CGF as a restoration material in bony defects.

Sufficient alveolar ridge dimensions is important for implant placement, it is also necessary that the regenerated bone is of good quality. It is pointed out that the quality and quantity of regenerated bone influence the initial stability of implant and can determine the success of dental implant osseointegration [38, 39]. The new bone density of CGF group was significantly higher than control group. This showed that the osteogenic effect of CGF combined with DBBM in patients with anterior tooth loss is better than using DBBM alone, especially in the early stage. Considering correlation between the bone quality and quantity, future studies about combining platelet concentrates and bone graft materials are needed.

Relaying on the strong soft tissue induction ability of CGF membrane [40], a full and sufficient keratinized gingival could be seen 3 months after the operation. The LWHI in the CGF group was higher than the control group 1 week after the operation, indicating that CGF promoted the rapid growth of soft tissue. It was worth mentioning that the VAS score of CGF group on the 1st day after operation was significantly lower than control group, indicating that CGF may relieve postoperative pain. In addition, we found the extraction sites of the two groups healed well after operation, but the attached gingiva in the CGF group were sufficient and plump, while the alveolar ridge in the control group atrophy in varying degrees and the attached gingiva were relatively narrow. The proportion of patients without bone grafting in the CGF group was significantly higher than the control group, suggesting that CGF combined with DBBM can reduce the proportion of patients with bone increment, reduce the cost of bone grafting, shorten the treatment period and create favorable conditions for implant and postoperative aesthetic effect.

## Conclusion

Although long-term studies with large samples are still needed, the following conclusions can be drawn. The use of CGF combined with DBBM in the anterior tooth region can help to reduce postoperative pain at the early stage of healing, form sufficient keratinized gingival tissue and effectively maintain the bone mass of alveolar bone in the three-dimensional direction. Meanwhile, it can provide good conditions for implant repair in the future, and reduce the need for bone grafting before implantation.

## Data Availability

The datasets generated and analyzed during the current study are not publicly available because they contain private medical information of patients, they agree to conduct the study and do not agree to disclose the raw data. However, it can be obtained from the corresponding author upon reasonable request.
